# Influenza vaccination in the Americas: Progress and challenges after the 2009 A(H1N1) influenza pandemic

**DOI:** 10.1080/21645515.2016.1157240

**Published:** 2016-05-19

**Authors:** Alba María Ropero-Álvarez, Nathalie El Omeiri, Hannah Jane Kurtis, M. Carolina Danovaro-Holliday, Cuauhtémoc Ruiz-Matus

**Affiliations:** Pan American Health Organization, Comprehensive Family Immunization Unit, Family, Gender and Life Course Department, Washington, DC, USA

**Keywords:** Americas, influenza vaccines, immunization, Latin America and the Caribbean, seasonal influenza

## Abstract

**Background**: There has been considerable uptake of seasonal influenza vaccines in the Americas compared to other regions. We describe the current influenza vaccination target groups, recent progress in vaccine uptake and in generating evidence on influenza seasonality and vaccine effectiveness for immunization programs. We also discuss persistent challenges, 5 years after the A(H1N1) 2009 influenza pandemic.

**Methods**: We compiled and summarized data annually reported by countries to the Pan American Health Organization/World Health Organization (PAHO/WHO) through the WHO/UNICEF joint report form on immunization, information obtained through PAHO's Revolving Fund for Vaccine Procurement and communications with managers of national Expanded Programs on Immunization (EPI).

**Results**: Since 2008, 25 countries/territories in the Americas have introduced new target groups for vaccination or expanded the age ranges of existing target groups. As of 2014, 40 (89%) out of 45 countries/territories have policies established for seasonal influenza vaccination. Currently, 29 (64%) countries/territories target pregnant women for vaccination, the highest priority group according to WHO´s Stategic Advisory Group of Experts and PAHO/WHO's Technical Advisory Group on Vaccine-preventable Diseases, compared to only 7 (16%) in 2008. Among 23 countries reporting coverage data, on average, 75% of adults ≥60 years, 45% of children aged 6–23 months, 32% of children aged 5–2 years, 59% of pregnant women, 78% of healthcare workers, and 90% of individuals with chronic conditions were vaccinated during the 2013–14 Northern Hemisphere or 2014 Southern Hemisphere influenza vaccination activities. Difficulties however persist in the estimation of vaccination coverage, especially for pregnant women and persons with chronic conditions. Since 2007, 6 tropical countries have changed their vaccine formulation from the Northern to the Southern Hemisphere formulation and the timing of their campaigns to April-May following the review of national evidence. LAC countries have also established an official network dedicated to evaluating influenza vaccines effectiveness and impact.

**Conclusion**: Following the A(H1N1)2009 influenza pandemic, countries of the Americas have continued their efforts to sustain or increase seasonal influenza vaccine uptake among high risk groups, especially among pregnant women. Countries also continued strengthening influenza surveillance, immunization platforms and information systems, indirectly improving preparedness for future pandemics. Influenza vaccination is particularly challenging compared to other vaccines included in EPI schedules, due to the need for annual, optimally timed vaccination, the wide spectrum of target groups, and the limitations of the available vaccines. Countries should continue to monitor influenza vaccination coverage, generate evidence for vaccination programs and implement social communication strategies addressing existing gaps.

## Background

Vaccination is the most effective measure to prevent influenza illness and its complications.^1^ Safe and effective vaccines are available^2^ and have been used for decades. Trivalent inactivated influenza virus vaccines (IIV) that contain 3 inactivated vaccine strains, including an influenza A/H1N1 strain, an influenza A/H3N2 strain, and an influenza B strain, are commonly used and are available as standard or high dose vaccines. Live attenuated virus vaccines (LAIV) are also available for use in healthy individuals only.^3,4^ Recently, quadrivalent vaccines that include 2 lineages of influenza B viruses have become available (as either IIV or LAIV vaccines). The World Health Organization (WHO) issues recommendations for vaccine composition twice a year: in February for a Northern Hemisphere vaccine and in September for a Southern Hemisphere vaccine.^5^

In 2012, WHO updated its position paper on immunization against influenza following the review of the epidemiological evidence available globally and recommendations from the Strategic Advisory Group of Experts (SAGE) on immunization.^6,7^ WHO recommended that countries considering the initiation or expansion of programs for seasonal influenza vaccination should include pregnant women as the highest priority group. Additionally, in no particular order of priority, the following high risk groups were recommended for vaccination: children aged 6–59 months (especially 6­23 months), the elderly, individuals with chronic medical conditions, and health care workers.^7^ These global recommendations were subsequently endorsed by the Pan American Health Organization (PAHO)'s Technical Advisory Group on vaccine-preventable diseases (TAG).^8^ PAHO is an international public health agency with more than 100 years of experience in working to improve health and living standards of the countries of the Americas. PAHO serves as the WHO Regional Office for the Americas, and provides technical assistance to all countries and territories in the Western Hemisphere. However, most of PAHO's technical cooperation is targeted to countries of Latin America and the Caribbean (LAC).^9^ During the 2009 A(H1N1) influenza pandemic, the Americas were one of the regions with the highest pandemic A(H1N1) vaccine uptake. LAC countries vaccinated over 145 million persons benefiting from the long history of seasonal vaccination and vaccination programs in place.^10^

We aimed to describe the progress of seasonal influenza vaccination programs in the Americas since the 2009 A(H1N1) influenza pandemic,^11^ including the vaccines currently used, the high risk groups targeted for vaccination, and the vaccination coverage for the 2013–14 Northern Hemisphere and 2014 Southern Hemisphere vaccination seasons. We also aimed to describe the advances of LAC countries in measuring vaccine effectiveness. Regarding influenza viruses seasonality and the best timing of vaccination campaigns and vaccines formulations to use, it is important to highlight that the great majority of LAC countries are located between the Tropic of Cancer and the Tropic of Capricorn. Contrarily to countries from temperate zones such as Argentina, Chile and Uruguay, where well-defined influenza seasons have allowed for an optimal planning of vaccination, tropical countries have historically had difficulties characterizing the seasonality of influenza viruses' circulation. Three sub-regions, namely the Southern Cone, the Andean region and Central America show virus circulation that is typical of the Southern Hemisphere (peak activity mid-year).^12,15^ (Supplemental Fig. 1). Finally, we summarized the remaining challenges for EPI programs that provide influenza vaccination in LAC countries, 5 years after the 2009 A(H1N1) influenza pandemic.

## Results

### Current policies and vaccination target groups in the Americas

As of 2014, 40 (89%) out of 45 countries/territories in the Americas have policies in place for vaccination against seasonal influenza. Since 2008 (pre-pandemic), 5 (11%) additional countries have introduced influenza vaccines in the public sector: Aruba, Bolivia, Dominica, Saint Vincent, and Suriname (Table 1). Various population groups are targeted by vaccination policies in the countries of the Americas, ranging from prioritized high risk groups to universal vaccination. The elderly are currently targeted for vaccination in 38 (84%) countries/territories, healthy children in 25 (56%) countries/territories, children suffering chronic/underlying conditions in 5 (11%) countries/territories, pregnant women in 29 (64%) countries/territories, healthcare workers in 38 (84%) countries/territories, and adults with chronic/underlying conditions in 35 (78%) countries/territories (Table 1). Since 2008, 25 (56%) countries/territories have included additional high risk groups for vaccination, 5 (11%) have expanded the age range for targeted elderly age groups (from 65 y old to 60 or 50 y old) and 1 (2%) country expanded the upper age range for children from 2 to 5 y of age. Note that chronic/underlying conditions may include: respiratory diseases (asthma, chronic bronchitis or emphysema), heart disease (atherosclerosis, cardiomyopathy/heart failure), neurodevelopmental disorders (cerebral palsy, muscular dystrophies, cognitive disorders), metabolic disorders (diabetes), immune system disorders (HIV/AIDS, chemotherapy, transplant patients or patients taking immunosuppressants, or chronic corticosteroid treatment), chronic renal failure in dialysis, chronic liver disease, especially cirrhosis, morbid obesity, blood diseases (sickle-cell disease, thalassemia major), and children on long-term aspirin therapy (risk of Reye's syndrome).

**Table 1.** Countries and Territories in the Americas with policies for seasonal influenza vaccination, 2004–2014. [Source: Official and unofficial reports to PAHO].

Number of countries with:	2004	2008	2014
Policies for influenza vaccination^*^	13 (29%)	35 (78%)	40 (89%)
Vaccination of healthy children	6 (13%)	22 (49%)	25 (56%)
Vaccination of children with chronic diseases	-	-	5 (11%)
Vaccination of the elderly	12 (27%)	33 (73%)	38 (84%)
Vaccination of persons with chronic diseases	9 (20%)	24 (53%)	35 (78%)
Vaccination of health care workers	3 (7%)	32 (71%)	38 (84%)
Vaccination of pregnant women	3 (7%)	7 (16%)	29 (64%)

* Data not collected from the French Departments (French Guiana, Guadeloupe, Martinique).

Pearson's correlation coefficient for linear regression using data for 11 years = 0.9, p = 0.0002.

### Influenza vaccines used and vaccination strategies in the Americas

In LAC countries, 4 trivalent IIV products are currently in use and provided free of charge through EPIs. No LAC country/territory has reported the use of LAIV or quadrivalent vaccines to PAHO/WHO to date. There are currently 8 seasonal trivalent influenza vaccines authorized for use in Canada, 7 of which are inactivated and one is a live attenuated influenza vaccine (LAIV).^4^ In addition, there are 2 quadrivalent inactivated vaccines (QIV) available. In the United States, there are currently 3 QIV and 9 trivalent IIV (including one high-dose vaccine) available for use, in addition to one LAIV vaccine for healthy individuals 2­49 y of age.^3^

**Figure 1.**
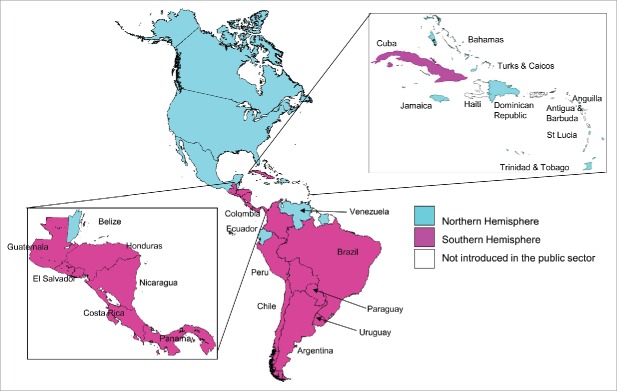
Use and formulation of seasonal influenza vaccines in the Americas, 2014.

Nine countries of the Americas use the Northern Hemisphere vaccine formulation and 31 use the Southern Hemisphere vaccine formulation administered mostly during April each year. (Fig. 1). In 2014, the majority of LAC countries (30 or 67%) purchased influenza vaccines through PAHO/WHO's Revolving Fund. LAC countries are in the process of building capacity for regional vaccine production. Argentina benefits from a private-public partnership for vaccine production and Brazil and Mexico have a transfer of vaccine technology agreement with vaccines manufacturers. Chile purchases influenza vaccines directly from manufacturers. In 2014, LAC countries acquired approximately 150 million doses of influenza vaccines.

Countries of the Americas use different vaccination strategies to prevent influenza, combining the offer of the vaccine through healthcare services and vaccination campaigns. Following vaccination campaigns, influenza vaccines continue to be offered through the routine health services throughout the influenza season until vaccines stock exhaust or expire. At least 14 countries in the Americas schedule short to medium-length intensive vaccination campaigns. For instance in 2014, Brazil administered over 36 million vaccine doses, reaching coverage of ∼90% of the target populations within a month. Other countries such as Argentina, Chile, the United States and Canada, organize longer “winter campaigns.” Considering influenza viruses circulation among most LAC countries is that of the Southern Hemisphere, countries take advantage of vaccination activities of the “Vaccination Week of the Americas (VWA)” initiative in late April that typically precedes the influenza seasons (Supplemental Fig. 1). During VWA in 2015, at least 14 countries/territories (Argentina, Barbados, Brazil, British Virgin Islands, Colombia, Costa Rica, El Salvador, Grenada, Guatemala, Honduras, Panama, St. Lucia, Uruguay and Venezuela) reported organizing some type of influenza-related activity.

### Influenza vaccination coverage in the Americas (2014)

Although 40 countries in the Americas have policies for seasonal influenza vaccination, not all of them report vaccination coverage estimates to PAHO/WHO systematically. Of 38 countries that target the elderly for vaccination, 23 (61%) reported vaccination coverage estimates to PAHO/WHO for 2014: 10 countries for adults ≥ 65 years, 9 countries for adults ≥ 60 years and 4 countries for adults ≥50 years. The median reported vaccination coverage for this group was 75% (Interquartile range (IQR) = 52%) (Table 2, Fig. 2a). Of 30 countries/territories that target children ≥ 6 months for vaccination, 20 (67%) reported vaccination coverage estimates to PAHO/WHO. The median vaccination reported coverage for children 6–23 months was 45% (IQR = 44%) and corresponded to a complete annual immunization schedule only i.e. 2 doses among vaccine-naive children and a single dose in previously vaccinated children (Table 2, Fig. 2b). The median vaccination reported coverage for children aged 2–5 y was 32%, with an interquartile range (IQR) of 62% (Table 2.). Of 29 countries/territories that target pregnant women for vaccination, 19 (66%) reported a vaccination coverage estimate to PAHO/WHO. The median vaccination reported coverage for this group was 59% (IQR = 54%) (Fig. 2c). Of 38 countries/territories that target healthcare workers for vaccination, 21 (75%) reported vaccination coverage estimates to PAHO/WHO. The median vaccination reported coverage for this group was 78% (IQR = 38%) (Fig. 2d). Of 35 countries/territories that target individuals with chronic diseases for vaccination, 14 (40%) reported a vaccination coverage estimate to PAHO/WHO. The median vaccination reported coverage was 90% (IQR = 27%).

**Table 2.** Overview of target influenza vaccination groups and coverages in the Americas, 2014. [Source: Official reports to PAHO].

	Children		Elderly		Other Risk Groups		
	6–23 months^(a)^	2–5 years	Schedule	Coverage	Healthcare workers	Persons with chronic conditions	Pregnant women
Anguilla	NA^*^	NA	≥50 y	…	yes	…	NA
Antigua and Barbuda	…	…	…	…	…	…	…
Argentina	50.4	no	≥65 y	90.2	84	35	96^(g)^
Aruba	…	…	≥60 y	…	…	…	…
Bahamas	11.3	yes	≥50 y	100	yes	yes	yes
Barbados	NA	NA	≥65 y	100	yes	…	NA
Belize	…	no	≥65 y	8.6	36	…	75
Bermuda	…	…	≥50 y	45.8	…	…	16
BES^**^	…	…	…	…	…	…	…
Bolivia	44.4	no	≥65 y	89	61	100^(c)^	59
Brazil	81.5	yes	≥60 y	86	97	yes	84
Canada	…	…	≥50 y	…	yes	yes	yes
Cayman Islands	…	yes	≥50 y	9.7	yes	73	3
Chile	72.2	no	≥65 y	75.03	100^(c)^	100^(c)^	97
Colombia	77.1	yes	≥50 y	100^(c)^	yes	yes	yes
Costa Rica	NA	NA	NA	NA	NA	NA	NA
Cuba	NA	…	≥65 y	100^(c)^	100^(c)^	92	88
Curaçao	…	…	≥60 y	…	…	…	…
Dominica	…	yes	≥65 y	100	60^(d)^	…	yes
Dominican Republic	1.7	no	≥65 y	6.3	42	yes	13
Ecuador	62.8	yes	≥60 y	39	100^(c)^	88	31
El Salvador	68.2	yes	≥60 y	74.9	78	…	75
Grenada	…	yes	≥50 y	…	yes	yes	NA
Guatemala	7.1	yes	≥65 y	64	100	74	17
Guyana	NA	NA	NA	NA	NA	NA	NA
Haiti	NA	NA	NA	NA	NA	NA	NA
Honduras	…^(b)^	…^(b)^	≥60 y	78.9	85	100^(c)^	NA
Jamaica	…^(b)^	…^(b)^	…	…	16^(c)^	20	…
Mexico	79.1	yes	≥60 y	90.6	92	100^(c)^	86
Montserrat	NA	NA	NA	NA	NA	NA	NA
Nicaragua	…^(b)^	…^(b)^	…	…^(b)^	68	100^(c)^	81
Panama	21.7	yes	≥60 y	68.8	79	yes	54
Paraguay	44.8	yes(32.3)	≥60 y	41.7	59	yes	79
Peru	36.5	no	≥60 y	89	97	100	30
St. Kitts and Nevis	NA	NA	NA	NA	NA	NA	…
St. Lucia	NA	NA	≥50 y	…	yes	yes	42
St. Vincent and the Grenadines	NA	NA	NA	NA	NA	NA	…
Sint Maarten^†^	…	…	…	…	…	…	…
Suriname	NA	NA	…	…	yes	yes	yes
Trinidad and Tobago	…	…	…	…	…	…	…
Turks and Caicos	23.7	yes (19.6)	≥50 y	…	yes	yes	yes
United States of America	…	…	≥50 y	…	yes	yes	yes
Uruguay	47.2	yes(81.9)	≥65 y	30.2	46	…	yes
Venezuela	24.1	yes	≥60 y	9.6	74^(f)^	10	34
Virgin Islands (UK)	NA	NA	NA	NA	5^(f)^	85	NA

* Not applicable.

** BES – Bonnaire, St. Eustatius, and Saba

† Sint Maarten using 50% of live-births in vaccination coverage calculations

(a) Formula used: Coverage = ((second dose + single dose)/Denominator)*100

(b) with chronic disease

(c) reported coverage >100%

(d) high refusal rates

(e) low intake of this vaccine by HCW

(f) denominator not updated since 2009

(g) 50% de NV

**Figure 2a.**
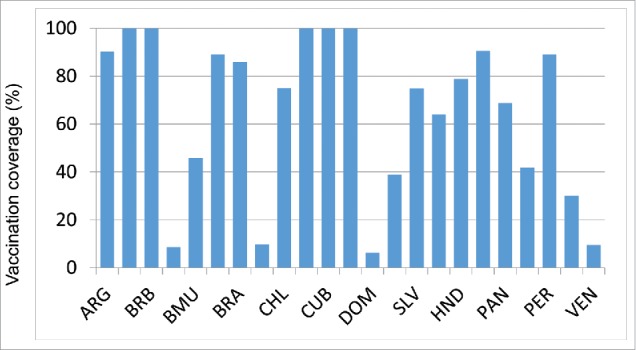
Influenza vaccination coverage among adults 60 years of age or older, data from selected countries, 2014. Note all countries used the administrative method for estimating influenza vaccination coverage.

**Figure 2b.**
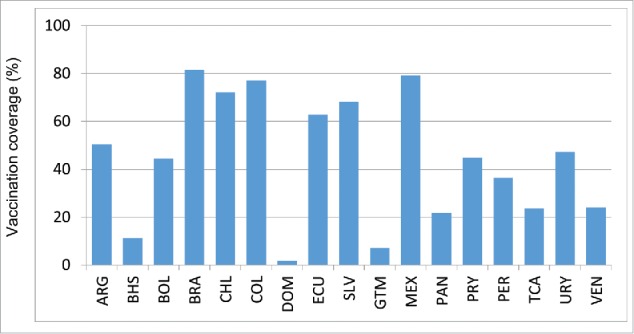
Influenza vaccination coverage among children 6–23 months, data from selected countries, 2014.

**Figure 2c.**
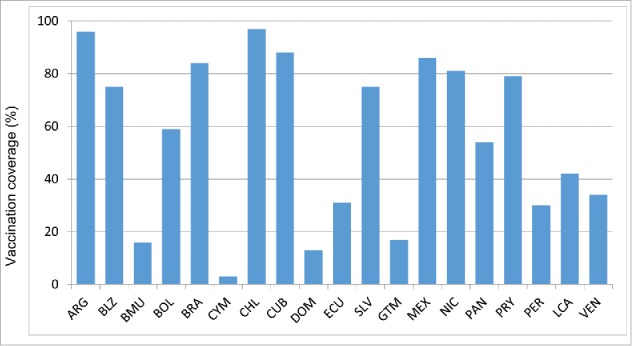
Influenza vaccination coverage among pregnant women, data from selected countries, 2014.

**Figure 2d.**
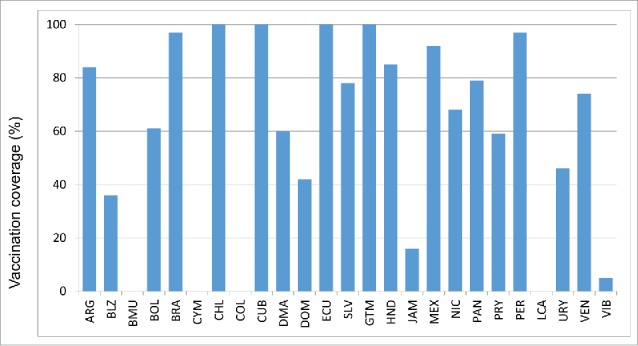
Influenza vaccination coverage among healthcare workers, data from selected countries, 2014.

### Progress in creating evidence to inform influenza vaccination programs in LAC countries

Countries of the Americas have been contributing to global influenza virological surveillance for decades. In 2004, PAHO's TAG recommended that LAC countries strengthen epidemiological and virological influenza surveillance in order to provide information on high-risk groups and help determine the best formulation and timing of vaccination for countries of the American tropics. Since then, LAC countries have made significant progress in the collection of timely and quality epidemiological data, as well as in laboratory confirmation and characterization of circulating influenza viruses.^13^ The scarcity of epidemiological information regarding severe influenza cases during the 2009 A(H1N1) influenza pandemic has also prompted many countries to invest in strengthening the surveillance of hospitalizations associated with influenza infections.^14^ As epidemiological and virological data became more available, 6 countries have changed their vaccine formulations from the Northern to the Southern Hemisphere formulation since 2007 (El Salvador, Guatemala, Colombia, Costa Rica, Cuba, and Honduras). These countries also changed the timing of vaccination to April–May. In all countries, the decision was based on a review of epidemiological and virological surveillance data by a multidisciplinary/inter-institutional committee (EPI, influenza surveillance, national influenza centers) and accompanied by the National Immunization Technical Advisory Groups (NITAGs). Central American countries collaborated and pooled their national antigenic characterization data into a regional analysis and confirmed that the Southern Hemisphere formulation corresponded to the most updated formulation available before the start of influenza seasons. The analysis of influenza surveillance data from 2002–2008 and 2011–2014 in the American tropics (101 y of cumulative data; 403,584 specimens tested) indicated that 13 (81%) of 16 participating countries had influenza epidemics that, on average, started during May and lasted 4 months.^12^ This regional analysis was a decisive element for the policy adjustments and for developing regional TAG recommendations in July 2015.^8^ In the Andean region, only Ecuador and Venezuela currently use the Northern Hemisphere vaccine (Fig. 1).

Regarding vaccines performance, since the mid-2000s, estimates of influenza vaccine effectiveness (interim and end-of-season estimates) have been regularly published by public health agencies in the United States and Canada.^16,17^ Despite substantial increases in vaccine uptake since 2004 in the rest of the Americas,^11^ such reports of vaccine effectiveness have been rare.^18-20^ In order to address this gap in evidence, a multicenter evaluation of influenza vaccine effectiveness against severe illness was set up in 2013 in LAC countries^13^ that led to the official establishment and launch of a network for influenza vaccine evaluations in the LAC region known as REVELAC-i (*Red para la Evaluación de Vacunas En Latino América y el Caribe–influenza*).^13^ This network is meant to support countries in generating and integrating evidence for influenza vaccination programs, including vaccine effectiveness and impact, in order to sustain investments in vaccination programs, and guide public health measures. As of July 2015, 14 countries have joined the network (Argentina, Brazil, Chile, Colombia, Costa-Rica, Cuba, Ecuador, El Salvador, Honduras, Mexico, Nicaragua, Panama, Paraguay, and Uruguay), 9 of which have collected and shared data during the 2013 and 2014 influenza seasons. Preliminary findings from the 2013 influenza season, suggested a reduction by approximately half of hospitalizations due to influenza among children 6­59 months (vaccine effectiveness [VE] = 43%; 95% confidence interval [95%CI]= 10%–64%) and adults ≥ 60 years (VE = 48%; 95%CI = 33%–59%). REVELAC-i has greatly benefitted from the recent improvements to EPI information systems across the region. Indeed, collecting data for vaccine effectiveness was less time-consuming and required fewer national resources in countries that had electronic national immunization registries in place such as Chile, Colombia and Costa Rica. It also boosted data quality of vaccination status recording across participating countries and made further use of SARI surveillance platforms.

### Influenza-related communication

As of 2014, all LAC countries carry out social communication campaigns to accompany vaccination activities. LAC countries have acquired significant experience in this domain especially in the use of public figures, champions (for instance football players in Brazil), and political leaders to advocate for influenza vaccines, among others. Nevertheless, more structured communication plans are needed to reach vulnerable populations such as pregnant women and individuals with chronic conditions. Moreover, communication to healthcare workers is crucial because they are key actors in recommending influenza vaccines to the public.

## Discussion

Since the 2009 A(H1N1) influenza pandemic, influenza vaccination programs across the region have not remained static. Influenza vaccination is challenging compared to other EPI vaccines because of the need for annual vaccination, for defining the best timing and formulation to use in tropical countries, the diversity in vaccination target groups across the life course, the generally moderate effectiveness of the available vaccines, and the vaccines' financial and related costs. Countries of the Americas have continued to progress in targeting high risk groups (5 new countries with policies for influenza vaccination, 25 countries/territories targeting new groups and 6 expanding the age range of existing groups), reflecting the different countries´ approaches in targeting those groups, driven by evidence of influenza illness morbidity, mortality and resources available. The most notable advance has been countries' expansion of vaccination to pregnant women, one of the most severely affected sub-populations during the 20009 A(H1N1) influenza pandemic.^21,22^ Indeed, LAC countries learned the value of vaccinating pregnant women during the 2009 A(H1N1) influenza pandemic and subsequently introduced vaccination more systematically for this group. During 2014, we observed relatively good vaccination coverage across the Americas. The highest reported vaccination coverage estimates corresponded to individuals with chronic conditions (90%) followed by healthcare workers (78%), the elderly (75%), children (32% among 6–23 months and 45% among 2–5 years) and pregnant women (59%). The high coverage among the elderly can be explained by the long tradition of vaccination of this age group against influenza and by WHO´s recommendation in 2003 of establishing a goal for vaccination of the elderly. Nevertheless, estimating vaccination coverage for the different target groups remains challenging. For instance, the definition of healthcare workers can vary between countries, from including only those who provide direct patient care to all health professionals (including administrative staff). For individuals with chronic conditions, estimating both numerators and denominators for vaccination coverage calculations is difficult. For the latter denominators, countries typically rely on estimates from health surveys, including an assessment of the prevalence of chronic conditions in the general population, but such surveys remain rare in LAC. For pregnant women, while capturing this subpopulation is relatively easy through health services´ antenatal care programs, it is difficult to estimate the number of pregnant women that will be exposed to influenza viruses during one season and countries use different approximations for denominators. Calculating influenza coverage for children, typically from 6 months to 2 y of age or to 5 y of age, benefits from the EPI mechanisms in place to document the administration of other vaccines (nominal recording of vaccination status from local to regional/national levels, common use of vaccination cards, etc.). Nevertheless, the information systems in place do not always allow for the distinction between the receipt of the first and the second dose of annual vaccine among children. As of 2014, many countries have started reporting vaccination coverage to PAHO/WHO based on the receipt of 2 vaccine doses among vaccine-naïve children ≤ 9 years.

Influenza vaccination campaigns are fairly dynamic and countries are currently working to refine the mechanisms for vaccination coverage reporting. Implementing adequate communication strategies will be key for countries, such as many in the Caribbean sub region, that often offer influenza vaccines universally to create greater demand. Among countries that focus on targeting high risk groups, boosting vaccination coverage will depend largely on effective communication strategies, the engagement of the scientific community and the proactive role of the health care personnel. Obtaining endorsements from professional societies, such as associations of obstetricians/gynecologists, infectious disease specialists, midwives, and NITAGs have proven to increase adherence to influenza vaccination among pregnant women [EPI communications, Argentina, Brazil].

The influenza A(H1N1)2009 influenza pandemic, gave influenza surveillance momentum among LAC countries, especially with regards to the surveillance of severe outcomes. It contributed to improving data completeness and quality across all surveillance systems and to the surge in influenza virus testing and antigenic and genetic characterization. In recent years, countries in the American tropics, especially in Central America, have used different data sources, and statistical methods to describe the seasonality of influenza viruses' circulation and have made adjustments to their vaccination policies accordingly. For all these countries, the Southern Hemisphere formulation corresponded to the most updated formulation available before the period of highest influenza activity. In order to maximize the effect of vaccines, vaccination efforts should indeed be timed to occur prior to the highest concentration of influenza cases in a country. Late vaccination may only have limited benefits, considering possible waning immunity and decreasing vaccine effectiveness as influenza viruses undergo antigenic changes.^23^ In July 2015, PAHO's TAG reviewed the available evidence gathered by influenza vaccination programs and encouraged countries to evaluate the impact of the recent policy changes and to carry out time series analyses to better understand influenza viruses seasonality including at the subnational level in large countries or countries with microclimates.^8^

With such a widespread use of seasonal influenza vaccines in the Americas, it was important that LAC countries start documenting vaccine effectiveness in preventing severe influenza associated illnesses in real-life settings in order to sustain their use. Influenza vaccine effectiveness can vary widely between seasons, due to factors such as the match between vaccine and circulating viruses' strains, the type of vaccine, prior exposure to influenza viruses and to influenza vaccines, as well as the health status of the vaccinee. Considering these yearly fluctuations in vaccine effectiveness, the evaluation of the impact of an influenza vaccination program would require information from various influenza seasons. The setup of the REVELAC-i network has provided an opportunity for countries to address this evidence gap and gather additional information that may further guide vaccination programs. It also contributed to promoting multidisciplinary work (EPI, surveillance and national influenza centers) and integration between the corresponding teams, institutions and information systems that are crucial for the sustainability of evidence generation.

## Methods

We compiled information that countries in the Americas reported in April 2014 to the Comprehensive Family Immunization Unit at PAHO/WHO headquarters using the WHO/UNICEF Joint Reporting Forms on Immunization (JRF). The JRF was adjusted in 2013 to standardize the reporting on the different seasonal influenza vaccination schedules used in the Americas. This tool emphasizes the importance of providing information on the coverage of 2 doses of vaccine among children aged less than 9 y and prompts the reporting of vaccination coverage for all other targeted subpopulations.^24^

We also gathered information from PAHO/WHO's Revolving Fund, the regional mechanism for bulk purchase of vaccines and supplies in place since 1979. We completed the information through communications with national Expanded Programs on Immunization (EPI). We did not include data from the French Departments in the Caribbean (French Guiana, Guadeloupe and Martinique) because the latter do not routinely report to PAHO/WHO. We collected information about the most recent vaccination target groups, their respective vaccination coverage, vaccine formulations used, timing and vaccination strategies. We compiled information from EPI managers about the use of surveillance and EPI data to generate evidence for immunization programs (personal communications). The majority of vaccination coverage estimates in LACs are calculated using the administrative method, dividing the number of doses of vaccine administered in a target group by the most recent census projection for that group.^25^ Vaccination coverage among children aged less than 2 y was calculated by summing up the receipt of 2 doses among vaccine-naïve children and a single dose among previously vaccinated children. We did not compile information on the use of influenza vaccines in the private sector, however the majority of influenza vaccines among LAC countries was provided through the EPIs.

## Conclusions

Since the A(H1N1)2009 influenza pandemic, the use of seasonal influenza vaccines has continued to increase in the Americas. Programs have expanded to new high risk groups, especially pregnant women. The progress of influenza surveillance, documentation of vaccination history as well as the strengthening of the corresponding information systems, have allowed LAC countries to produce useful information that addresses the region's gap in knowledge. Several countries have already made adjustments to their policies accordingly. EPI programs are faced with additional technical and operational difficulties with influenza vaccination compared to other vaccines of EPI schedules, such as the need for annual vaccination, optimal timing of vaccination activities, and adequate priming of vaccine-naïve children with 2 doses. Influenza vaccination targets a wide spectrum of high-risk sub-populations, and can be costly for low to middle-income countries. Current efforts to measure vaccines performance and impact, complemented by diseases burden and economic studies may help health authorities sustain investments in influenza vaccines. In addition to preventing disease burden, comprehensive seasonal influenza vaccination activities (including vaccine procurement, the diversity of the high-risk groups, communication, technical and operational aspects) is the best way to prepare for a future influenza pandemic.

## Supplementary Material

KHVI_A_1157240_SupplementalFig.pdf
