# Supplementary material for: Influenza vaccination in the Americas: Progress and challenges after the 2009 A(H1N1) influenza pandemic
Source: Hum Vaccin Immunother. 2016 May 19;12(8):2206–14. doi: 10.1080/21645515.2016.1157240 (PMC4994725; doi:10.1080/21645515.2016.1157240)

Supplemental Figure 1. Influenza circulation by sub-region of the Americas, 2012–15. [source: [PAHO regional update, influenza].](PAHO regional update, influenza)

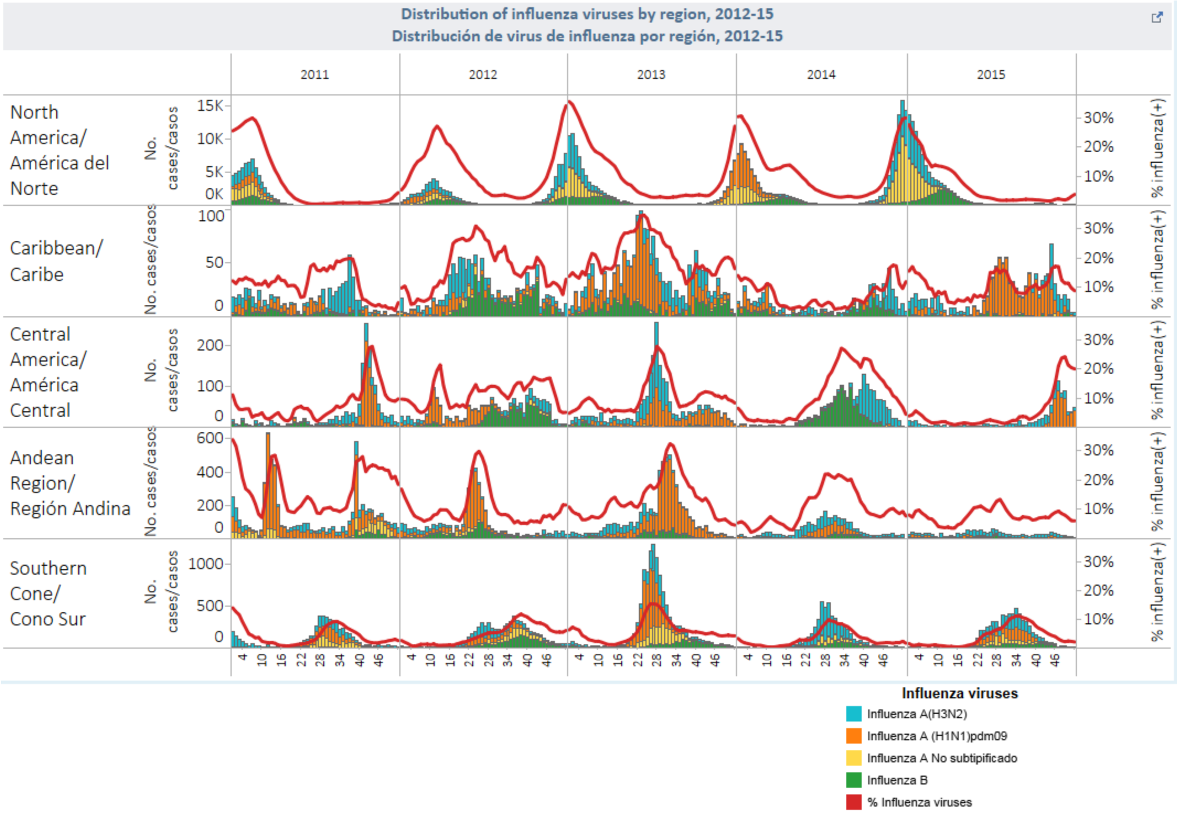

Supplement: KHVI_A_1157240_SupplementalFig.pdf [file khvi-12-08-1157240-s001.pdf]
